# Supplementary material for: Molecular basis of the reaction mechanism of the methyltransferase HENMT1
Source: PLoS One. 2024 Jan 10;19(1):e0293243. doi: 10.1371/journal.pone.0293243 (PMC10781085; doi:10.1371/journal.pone.0293243)
Supplement: S1 File — (PDF) [file pone.0293243.s001.pdf]

Micro RNAs

Micro RNAs (miRNAs) are short non-coding RNA molecules, about 21-24 nucleotides (nt) in length, that regulate gene translation through base-pairing complementary to silence or degrade target mRNAs. They are associated with almost every aspect of biological processes, including cell growth, metabolism, inflammation, apoptosis, and the pathophysiology of many diseases [1]. Recent studies have suggested circulating miRNAs play oncogenic roles and can serve as promising diagnostic and prognostic biomarkers for many diseases [2–10].

Hsa-miR-21-5p, as the first-identified miRNA molecule, is a representative, circulating, and typical onco-miRNA [2,7]. It has been extensively investigated in various malignancies, notably in brain cancer, lung cancer, colorectal cancer, pancreatic cancer, breast cancer, gastric cancer, esophageal cancer, and hepatocellular carcinoma [2,3,5,7,11]. Hsa-miR-21-5p post-transcriptionally regulates the expression of multiple cancer-related target genes, such as the phosphatase and tensin homolog (PTEN), the programmed cell death protein 4 (PDCD4), the reversion-inducing cysteine-rich protein with Kazal motifs (RECK), and the signal transducer activator of transcription 3 (STAT3) [12]. Hsa-miR-21-5p overexpression promotes tumor growth, metastasis and invasion, reduces sensitivity to chemotherapy, and is associated with poor survival in various cancers [11,13,14].

The Water Flooding (WF) Approach

It has been challenging to determine the internal water molecules within the active site due to the charges in the protein interior [16]. Warshel and co-workers have developed the water flooding approach which takes advantage of the Monte Carlo (MC). In this method, first, an excessive amount of internal water molecules were added to over-saturate the protein, and a postprocessing MC was used to evaluate and keep the most realistic configuration of the internal water molecules [17]. The development, and application of this water flooding approach have been described extensively, and it has become an efficient way to determine the most realistic configurations of water within protein [17–19]. Here we briefly list the key parameters we used in this study: In our simulation, the SCAAS surface constraints and the local reaction field (LRF) long-range treatment as well as polarizable ENZYMIX force field were used, and 10,000 steps of minimization followed by 200ps MD relaxation were done on our initial structure with a time step of 1.0 fs. The final structures were then used for WF simulations. During the WF simulations, a spherical hard wall was placed to prevent the exchange of the inside water molecules with the outside water molecules. The radius of the spherical hard wall for the cavity was set at 6.0 Å.

HENMT1 dependency in different cancer types revealed by DepMap

Over the last decades, large-scale endeavors and initiatives have been taken to systematically search for biomarkers by identifying genetic and pharmacological dependence of these cancer cells. One of them is the Dependency Map (DepMap), which uses genome-wide CRISPR and RNAi loss-of-function screens in 1086 cell lines to identify essential genes for proliferation. By exploring the latest progress in DepMap project (DepMap 22Q2 Public+Score, Chronos), we analyzed the gene effect and found that HENMT1 has gene effect greater than -0.5 [see Figure S6(A)], which indicates it is not an essential genes in the majority of cancer cell lines. Note that the essentiality of each gene was scored with 0 representing non-essential genes while −1 corresponding to the median score of all common essential genes. The HENMT1 dependent cell lines (58/1086) were analyzed based on the primary disease and are shown in Figure S6(B). Several lineages are represented including breast cancer (7), lung cancer (7), and skin cancer (6). It also shows that HENMT1 is related to the Breast Ductal Carcinoma or Carcinoma. The connection of HENMT1 in Lung Cancer has limited studies, 4/7 are in the Non-Small Cell Lung Cancer (NSCLC). Meanwhile, HENMT1 was categorized as "strongly selective" by DepMap CRISPR screening which means its dependency is at least 100 times more likely to have been sampled from skewed distribution than a normal distribution. Above all, targeting HENMT1 may exert a strong reduction of viability in a very small subset of cell lines [see Figure S6(C)], possible in the Acute Myelogenous Leukemia (AML), Carcinoma, Renal Carcinoma, and Multiple Myeloma, and these selective dependencies may associate with molecular features that can be explored as biomarkers.

Using STRING, which is based on the known and predicted protein-protein associations, integrated and transferred across organisms, we have done an in-depth study of the genes that have functional and physical associations with HENMT1 [see Figure S6(D)]. Among these genes, it shows a strong co-expression profile with HENMT1. Further analyzing these genes in disease subtype and lineage subsubtype reveals complex relationships across the lineages and different gender groups.

REFERENCES

1. Saliminejad K, Khorram Khorshid HR, Soleymani Fard S, Ghaffari SH. An overview of microRNAs: Biology, functions, therapeutics, and analysis methods. J Cell Physiol. 2019;234: 5451–5465. doi:10.1002/jcp.27486

2. Kao H-W, Pan C-Y, Lai C-H, Wu C-W, Fang W-L, Huang K-H, et al. Urine miR-21-5p as a potential non-invasive biomarker for gastric cancer. Oncotarget. 2017;8: 56389–56397. doi:10.18632/oncotarget.16916

3. Shi R, Wang P-Y, Li X-Y, Chen J-X, Li Y, Zhang X-Z, et al. Exosomal levels of miRNA-21 from cerebrospinal fluids associated with poor prognosis and tumor recurrence of glioma patients. Oncotarget. 2015;6: 26971–26981. doi:10.18632/oncotarget.4699

4. Peng Q, Zhang X, Min M, Zou L, Shen P, Zhu Y. The clinical role of microRNA-21 as a promising biomarker in the diagnosis and prognosis of colorectal cancer: a systematic review and meta-analysis. Oncotarget. 2017;8: 44893–44909. doi:10.18632/oncotarget.16488

5. Khalid U, Ablorsu E, Szabo L, Jenkins RH, Bowen T, Chavez R, et al. MicroRNA-21 (miR-21) expression in hypothermic machine perfusate may be predictive of early outcomes in kidney transplantation. Clin Transplant. 2016;30: 99–104. doi:10.1111/ctr.12679

6. Elbehidy RM, Youssef DM, El-Shal AS, Shalaby SM, Sherbiny HS, Sherief LM, et al. MicroRNA-21 as a novel biomarker in diagnosis and response to therapy in asthmatic children. Mol Immunol. 2016;71: 107–114. doi:10.1016/j.molimm.2015.12.015

7. Olivieri F, Spazzafumo L, Bonafè M, Recchioni R, Prattichizzo F, Marcheselli F, et al. MiR-21-5p and miR-126a-3p levels in plasma and circulating angiogenic cells: relationship with type 2 diabetes complications. Oncotarget. 2015;6: 35372–35382. doi:10.18632/oncotarget.6164

8. Migita K, Komori A, Kozuru H, Jiuchi Y, Nakamura M, Yasunami M, et al. Circulating microRNA Profiles in Patients with Type-1 Autoimmune Hepatitis. PLoS One. 2015;10: e0136908. doi:10.1371/journal.pone.0136908

9. Zendjabil M, Favard S, Tse C, Abbou O, Hainque B. [The microRNAs as biomarkers: What prospects?]. C R Biol. 2017;340: 114–131. doi:10.1016/j.crvi.2016.12.001

10. Armand-Labit V, Pradines A. Circulating cell-free microRNAs as clinical cancer biomarkers. Biomol Concepts. 2017;8: 61–81. doi:10.1515/bmc-2017-0002

11. He J-H, Li Y-G, Han Z-P, Zhou J-B, Chen W-M, Lv Y-B, et al. The CircRNA-ACAP2/Hsa-miR-21-5p/ Tiam1 Regulatory Feedback Circuit Affects the Proliferation, Migration, and Invasion of Colon Cancer SW480 Cells. Cell Physiol Biochem. 2018;49: 1539–1550. doi:10.1159/000493457

12. Tse J, Pierce T, Carli ALE, Alorro MG, Thiem S, Marcusson EG, et al. Onco-miR-21 Promotes Stat3-Dependent Gastric Cancer Progression. Cancers (Basel). 2022;14: 264. doi:10.3390/cancers14020264

13. Jiang J, Wang X, Lu J. PWRN1 Suppressed Cancer Cell Proliferation and Migration in Glioblastoma by Inversely Regulating hsa-miR-21-5p. Cancer Manag Res. 2020;12: 5313–5322. doi:10.2147/CMAR.S250166

14. Yu W, Zhu K, Wang Y, Yu H, Guo J. Overexpression of miR-21-5p promotes proliferation and invasion of colon adenocarcinoma cells through targeting CHL1. Molecular Medicine. 2018;24: 36. doi:10.1186/s10020-018-0034-5

15. Li H, Robertson AD, Jensen JH. Very fast empirical prediction and rationalization of protein pKa values. Proteins. 2005;61: 704–721. doi:10.1002/prot.20660

16. Bhattacharjee N, Biswas P. Structure of hydration water in proteins: a comparison of molecular dynamics simulations and database analysis. Biophys Chem. 2011;158: 73–80. doi:10.1016/j.bpc.2011.05.009

17. Chakrabarty S, Warshel A. Capturing the energetics of water insertion in biological systems: The water flooding approach. Proteins. 2013;81: 93–106. doi:10.1002/prot.24165

18. Zhao LN, Kaldis P. Cascading proton transfers are a hallmark of the catalytic mechanism of SAM-dependent methyltransferases. FEBS Letters. 2020;594: 2128–2139. doi:10.1002/1873-3468.13799

19. Yoon H, Kolev V, Warshel A. Validating the Water Flooding Approach by Comparing It to Grand Canonical Monte Carlo Simulations. J Phys Chem B. 2017;121: 9358–9365. doi:10.1021/acs.jpcb.7b07726

20. Huang Y, Ji L, Huang Q, Vassylyev DG, Chen X, Ma J-B. Structural insights into mechanisms of the small RNA methyltransferase HEN1. Nature. 2009;461: 823–827. doi:10.1038/nature08433

21. Yu B, Yang Z, Li J, Minakhina S, Yang M, Padgett RW, et al. Methylation as a Crucial Step in Plant microRNA Biogenesis. Science. 2005;307: 932–935. doi:10.1126/science.1107130

22. Yang Z, Ebright YW, Yu B, Chen X. HEN1 recognizes 21-24 nt small RNA duplexes and deposits a methyl group onto the 2' OH of the 3' terminal nucleotide. Nucleic Acids Res. 2006;34: 667–675. doi:10.1093/nar/gkj474

23. Kirino Y, Mourelatos Z. The mouse homolog of HEN1 is a potential methylase for Piwi-interacting RNAs. RNA. 2007;13: 1397–1401. doi:10.1261/rna.659307
