# Supplementary figures and images for: Molecular basis of the reaction mechanism of the methyltransferase HENMT1
Source: PLoS One. 2024 Jan 10;19(1):e0293243. doi: 10.1371/journal.pone.0293243 (PMC10781085; doi:10.1371/journal.pone.0293243)

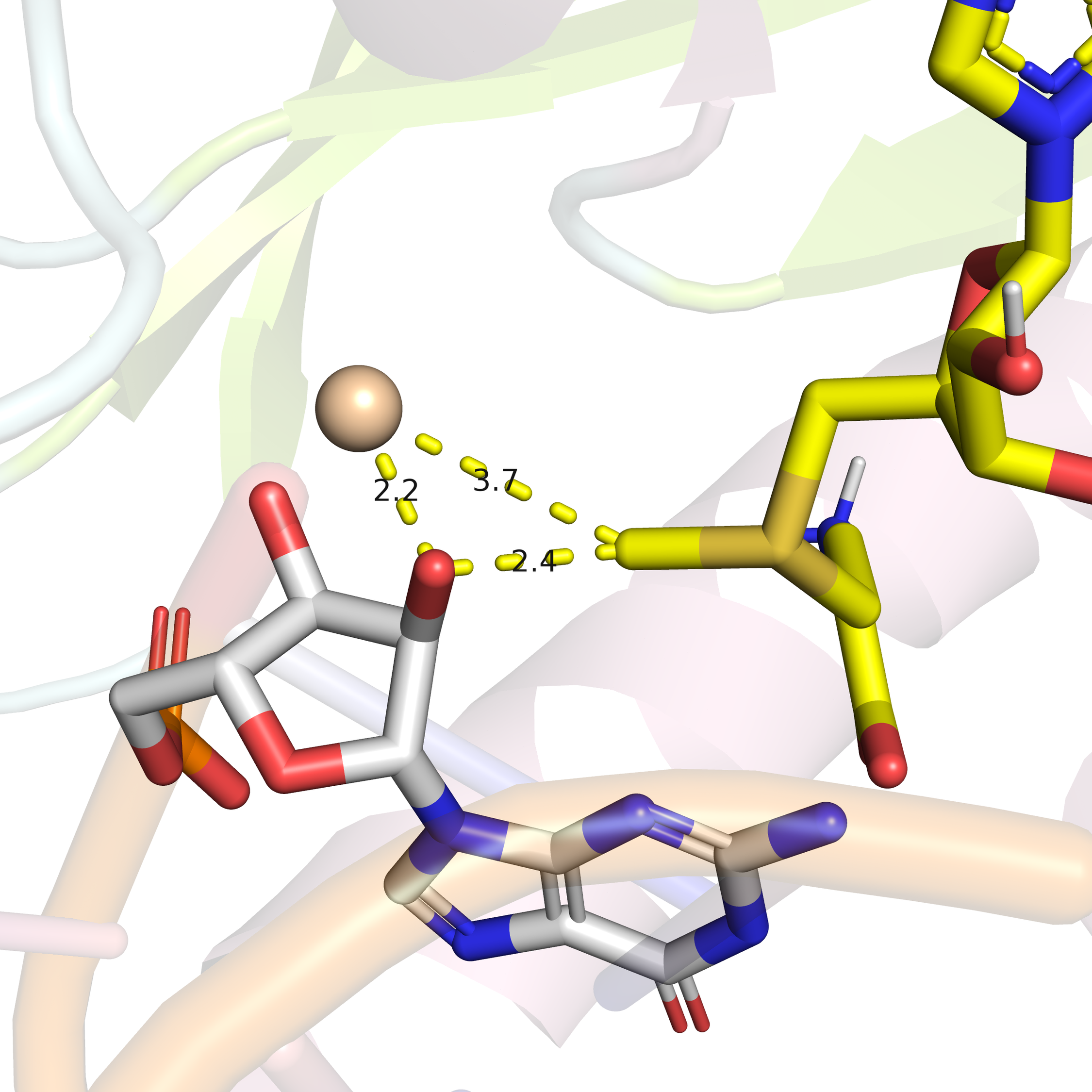

Supplement: S1 Fig — (TIF) [file pone.0293243.s002.tif]

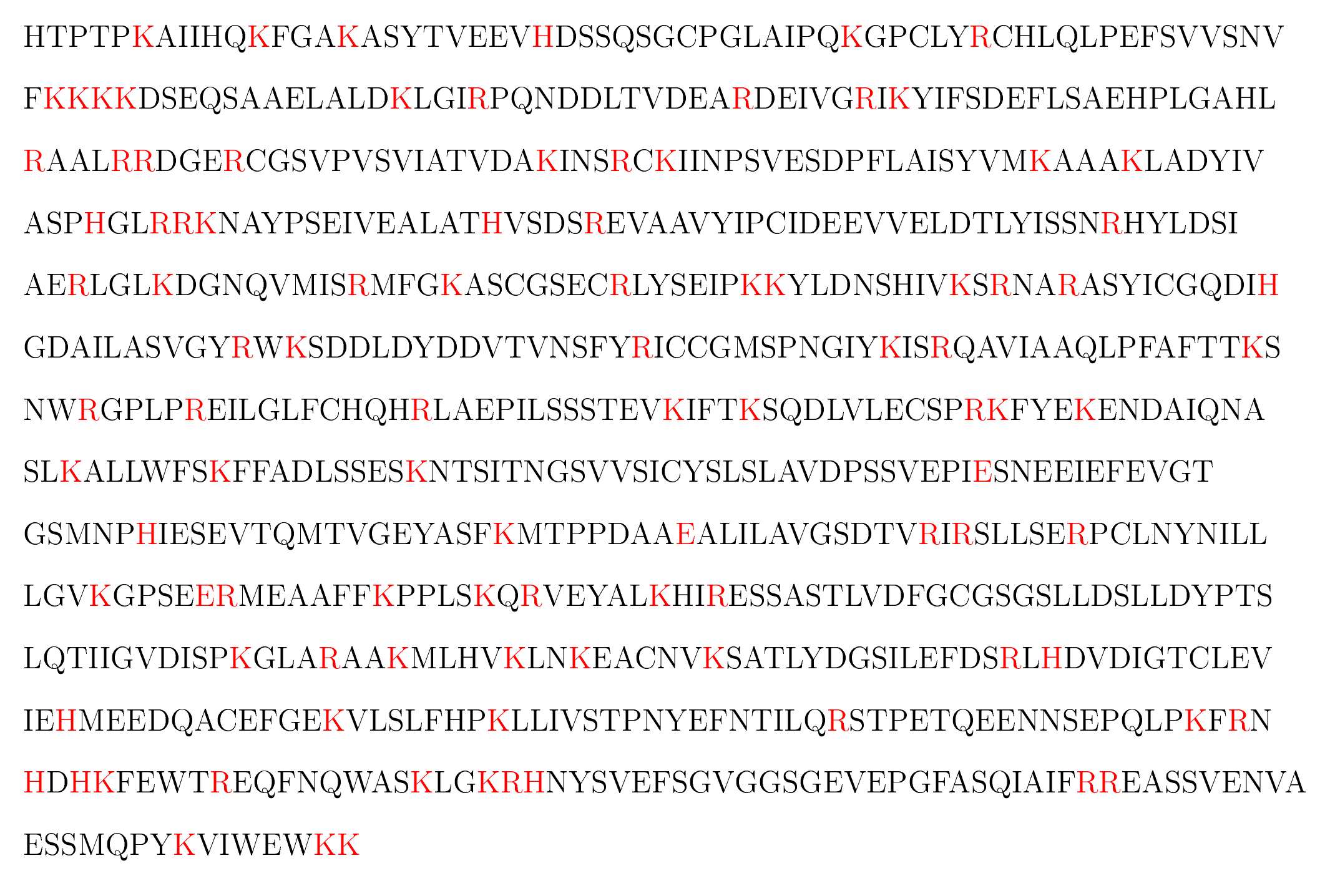

Supplement: S2 Fig — Note that protonated residues are shown in red. (TIF) [file pone.0293243.s003.tif]

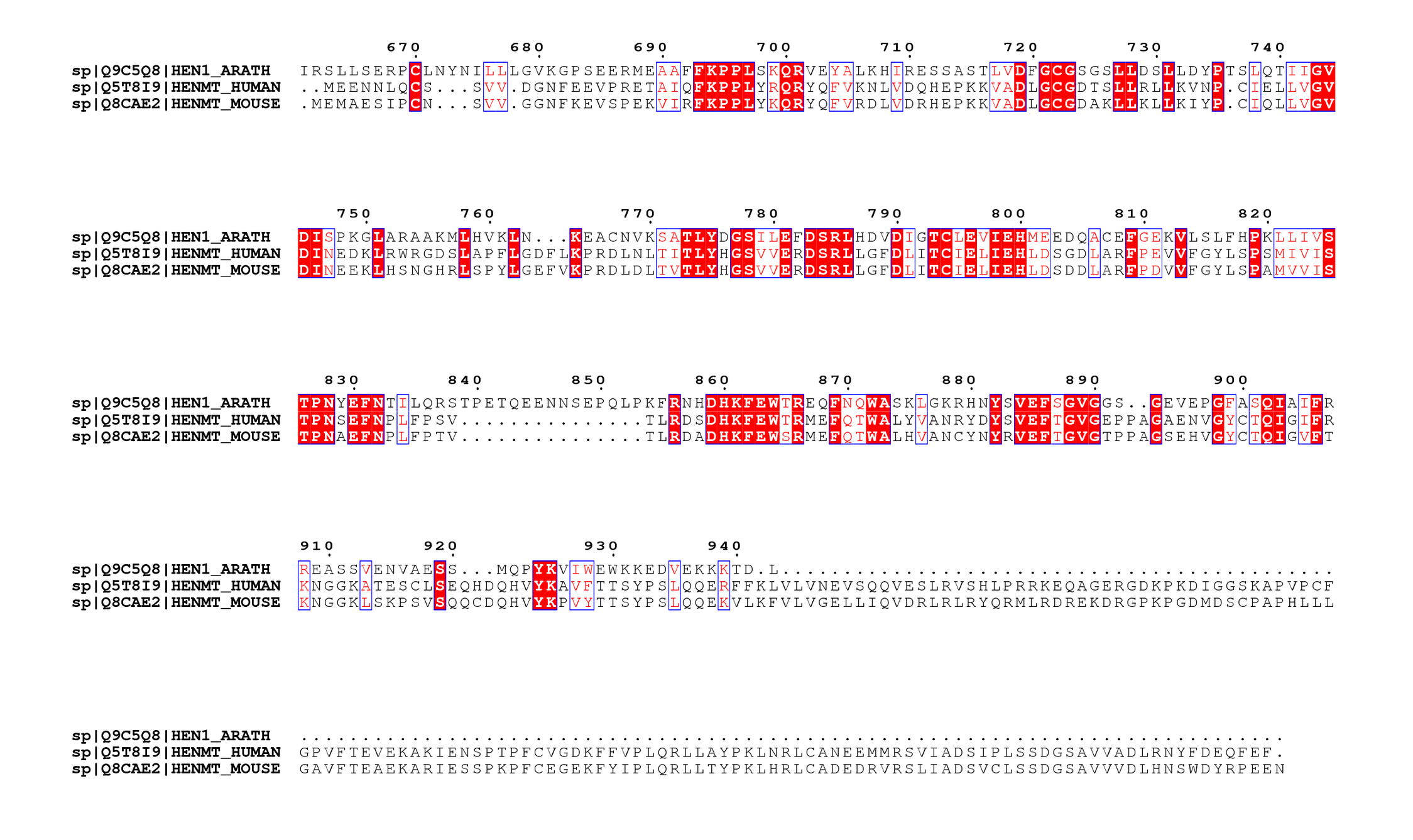

Supplement: S3 Fig — The alignment was done using Clustal Omega. This figure is generated using ESPript 3.x. (TIF) [file pone.0293243.s004.tif]

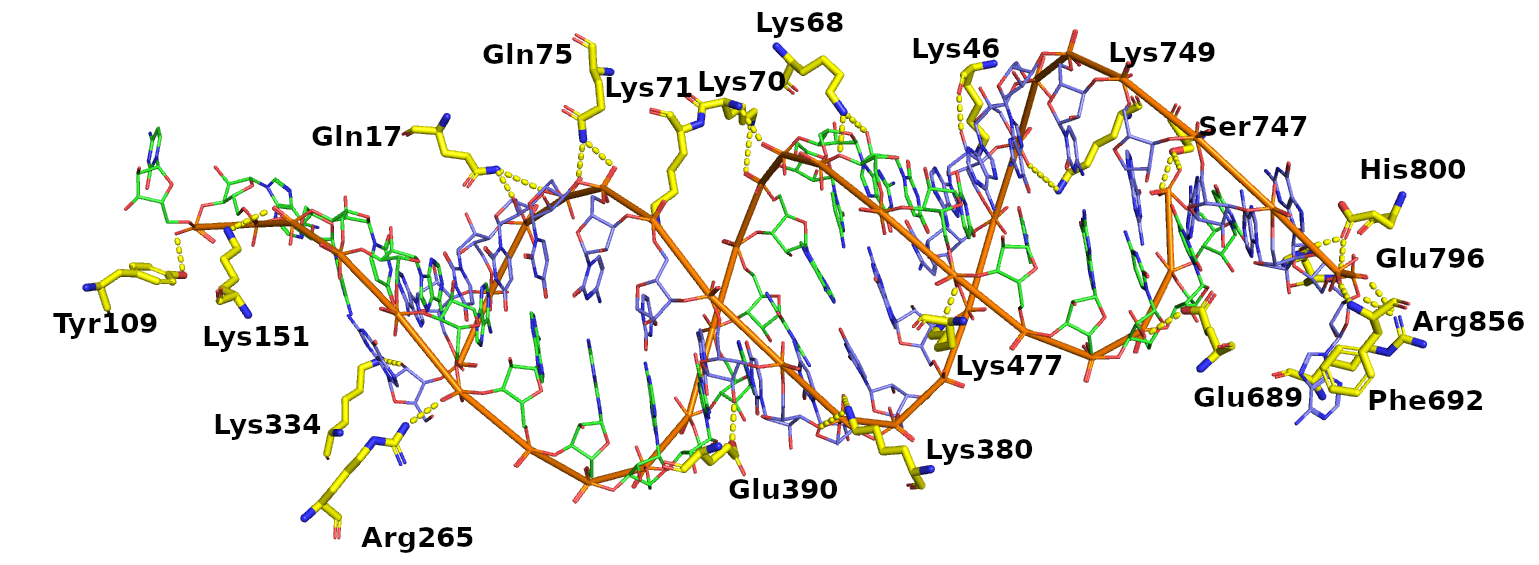

Supplement: S4 Fig — The residues from plant Hen1 are shown as stick and colored yellow for C atom. The backbone of miR173/miR173* are shown as ribbon and the ribose group and base are shown as lines. This figure is generated by PyMol. (TIF) [file pone.0293243.s005.tif]

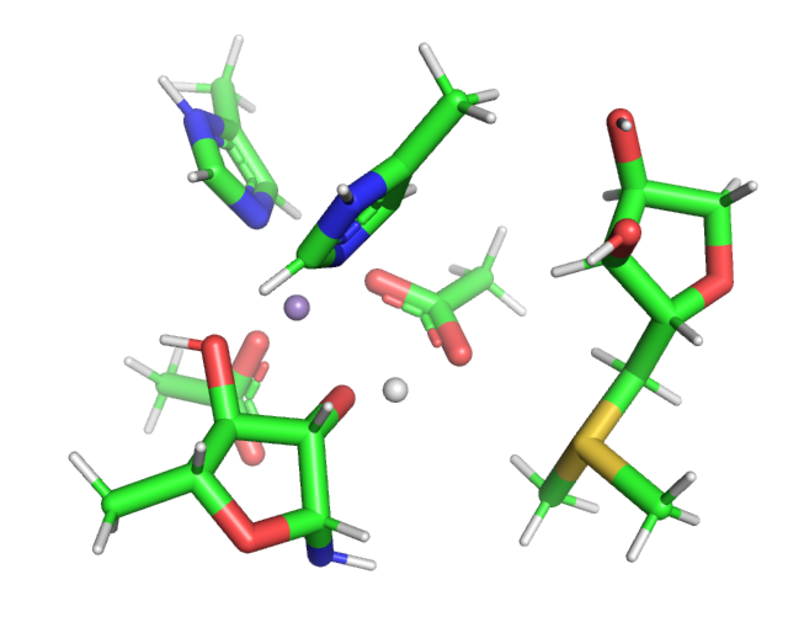

Supplement: S5 Fig — The white sphere is the hydrogen shared between 2’-O and Glu796. (TIF) [file pone.0293243.s006.tif]

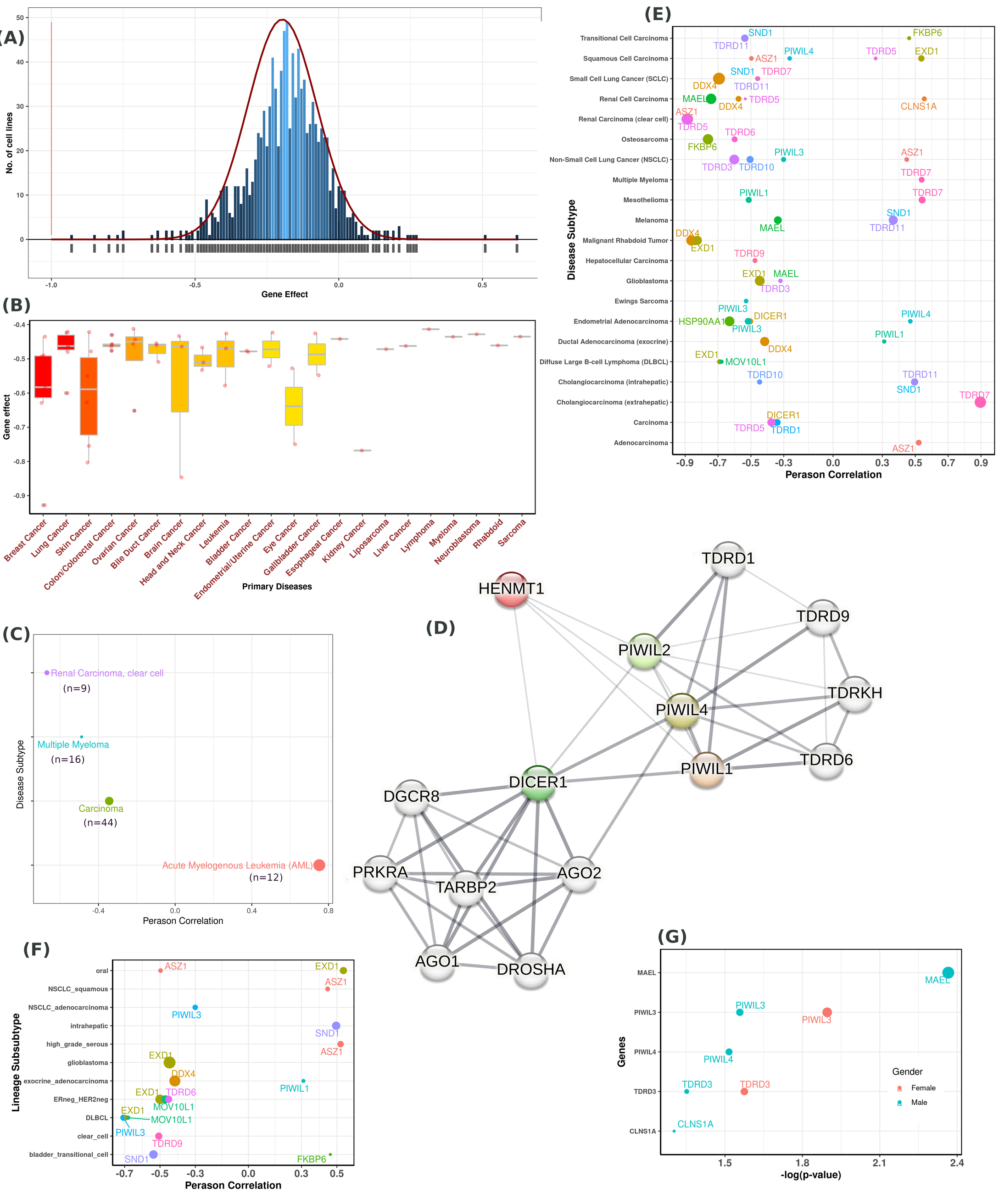

Supplement: S6 Fig — (A) Score distribution of HENMT1 in the genome-scale CRISPR-Cas9 screen. The x-axis represents the gene effect scores while the y axis represents the cell line distribution. Individual scores (DepMap 22Q2 Public+Score, Chronos) are indicated by the symbols depicted below the x-axis. (B) HENMT1 gene effect across different cancer types. (C) The correlation between HENMT1 gene effect (CRISPR DepMap 22Q2 Public) and its expression (Expression 22Q2 Public). The size indicates the -log10(p-value). The bigger the size corresponds to higher statistical significance. The number of points/samples are indicated in the bracket. (D) The predicted HENMT1 network, the thickness of the edge indicates the confidence score. This figure is generated using the String web server; the gene association network is predicted by STRING. (E) The correlation between HENMT1 gene effect and the expression of its associated genes based on the disease subtype and lineage subsubtype (F). (G) The expression of HENMT1 and the expression of the associated genes with statistical significance in male and female groups. (TIF) [file pone.0293243.s007.tif]
