# Supplementary material for: Molecular basis of the reaction mechanism of the methyltransferase HENMT1
Source: PLoS One. 2024 Jan 10;19(1):e0293243. doi: 10.1371/journal.pone.0293243 (PMC10781085; doi:10.1371/journal.pone.0293243)
Supplement: S1 Table — (PDF) [file pone.0293243.s008.pdf]

S1 Table: Calculated pKa for HEN1 with (w/) and without (w/o) miRNA using PropKa [15].

pK_a	Glu796	Glu799	His800	His860
w/o miRNA and w/ SAM	1.89	6.42	8.35	8.74
w/ miRNA and w/ SAH	1.07	7.74	10.22	5.30
