# Supplementary material for: Molecular basis of the reaction mechanism of the methyltransferase HENMT1
Source: PLoS One. 2024 Jan 10;19(1):e0293243. doi: 10.1371/journal.pone.0293243 (PMC10781085; doi:10.1371/journal.pone.0293243)
Supplement: S2 Table — (PDF) [file pone.0293243.s009.pdf]

S1 Table: Calculated summary of the partial charge during methyl transfer.

Distance	Mg	SAM	SAM Exclude $-CH_3$	Substrate	Substrate Include $-CH_3$	$-CH_3$
-1.07	0.585	0.719	0.831	-1.263		0.112
-0.99	0.585	0.717	0.830	-1.262		0.113
-0.91	0.586	0.714	0.828	-1.261		0.113
-0.83	0.588	0.711	0.825	-1.260		0.114
-0.74	0.589	0.707	0.822	-1.259		0.114
-0.66	0.590	0.704	0.820	-1.258		0.115
-0.58	0.591	0.701	0.818	-1.256		0.116
-0.50	0.592	0.698	0.815	-1.255		0.118
-0.42	0.594	0.694	0.813	-1.254		0.119
-0.34	0.595	0.690	0.810	-1.252		0.120
-0.25	0.597	0.685	0.806	-1.250		0.121
-0.17	0.599	0.678	0.800	-1.248		0.122
-0.09	0.601	0.657	0.788	-1.242		0.131
-0.01	0.606	0.607		-1.226		0.155
0.07	0.612	0.538		-1.200		0.186
0.15	0.619	0.457		-1.166		0.219
0.24	0.628	0.370		-1.127		0.251
0.32	0.637	0.281		-1.085		0.280
0.40	0.646	0.198		-1.043		0.305
0.48	0.654	0.124		-1.004		0.325
0.56	0.660	0.063		-0.970		0.339
0.64	0.664	0.017		-0.941		0.346
0.72	0.667	-0.016		-0.915		0.345
0.81	0.669	-0.036		-0.891		0.335
0.89	0.670	-0.048		-0.868	-0.550	0.318
0.97	0.671	-0.052		-0.854	-0.549	0.304
1.05	0.672	-0.053		-0.849	-0.552	0.297
1.13	0.673	-0.053		-0.847	-0.555	0.292
1.21	0.675	-0.053		-0.845	-0.557	0.288
1.30	0.675	-0.053		-0.844	-0.558	0.286
1.38	0.676	-0.053		-0.842	-0.557	0.285
1.46	0.676	-0.053		-0.840	-0.556	0.285
1.54	0.675	-0.053		-0.839	-0.554	0.284
1.62	0.675	-0.053		-0.837	-0.553	0.284
1.70	0.675	-0.053		-0.835	-0.552	0.283
1.79	0.675	-0.053		-0.834	-0.551	0.282
1.87	0.675	-0.052		-0.833	-0.551	0.282
