# Supplementary material for: Molecular basis of the reaction mechanism of the methyltransferase HENMT1
Source: PLoS One. 2024 Jan 10;19(1):e0293243. doi: 10.1371/journal.pone.0293243 (PMC10781085; doi:10.1371/journal.pone.0293243)
Supplement: S3 Table — The sequence and structure used for S1 and SII are miR173/miR173* from 3HTX.pdb. Note that the to-be-methylated strand is colored blue with the methyl 3’-end colored red. (PDF) [file pone.0293243.s010.pdf]

S3 Table: The sequence of the small RNA duplex (derived from one natural substrate of HEN1, termed miR173/miR173) substrate. The sequence and structure used for S1 and SII are miR173/miR173* from 3HTX.pdb. Note that the to-be-methylated strand is colored blue with the methyl 3'-end colored red.*

Sequence of plant Hen1 small RNA duplex substrate	SI	1 5 10 15 22<br>miR173 5'– U U C G C U U G C A G A G A G A A A U C A C –3'<br>| | | | | | | | | | | | | | | | | | | | | miR173* 3'– G A A A G C G A A C G U C U C U C U U U A G –5'<br>22 20 15 10 5 1
	SII	1 5 10 15 22<br>miR173 5'– U U C G C U U G C A G A G A G A A A U C A C –3'<br>| | | | | | | | | | | | | | | | | | | | | miR173* 3'– G A A A G C G A A C G U C U C U C U U U A G –5'<br>22 20 15 10 5 1
Sequence of mouse HEN1		1 5 10 15 20 20 28 30<br>piR-1 (WT) 5'–P-UGACAUGAAC ACAGGUGCUC AGAUAGCUUU–3'<br>piR-3 (WT) 5'–P-UGAGAGUGGC AUCUAAAUGU UUAGUGGU–3'<br>piR-3 (28A) 5'–P-UGAGAGUGGC AUCUAAAUGU UUAGUGGA–3'<br>piR-3 (28C) 5'–P-UGAGAGUGGC AUCUAAAUGU UUAGUGGC–3'<br>piR-3 (28G) 5'–P-UGAGAGUGGC AUCUAAAUGU UUAGUGGG–3'<br>The methylation efficiencies for piR-3 are: A (259%) > C (137%) > U (100%) > G(44%) [23]
